# COMBINED MULTIMODAL EXERCISE AND COGNITIVE TRAINING FOR PERSONS WITH MILD DEMENTIA: A RANDOMIZED CONTROLLED TRIAL

**DOI:** 10.1093/geroni/igae098.2265

**Published:** 2024-12-31

**Authors:** Dandan Xue, Polly Wai Chi Li, Doris Sau Fung Yu

**Affiliations:** The University of Hong Kong, Hong Kong, China (People’s Republic); The University of Hong Kong, Hong Kong, China (People’s Republic); The University of Hong Kong, Hong Kong, China (People’s Republic)

## Abstract

The effects of combined exercise and cognitive interventions among persons with mild dementia (PwMD) are not known. This randomized controlled trial randomized 72 PwMD to a caregiver-assisted combined multimodal exercise and cognitive training (CA-MECT) intervention or health education. The 12-week CA-MECT included online multimodal exercise and cognitive training through videoconferencing supplemented with home visits and home-based training. Caregivers assisted PwMD in intervention delivery and home-based training. Validated tools were used to evaluate cognitive, psychological, functional outcomes, and health-related quality of life (HRQoL). Compared to the control group, the intervention group showed significantly greater improvement in global cognition (β = 2.468, 95% CI [1.254, 3.681], p < 0.001), immediate recall (β = 2.924, 95% CI [1.387, 4.461], p < 0.001), short-term delayed recall (β = 0.63, 95% CI [0.005, 1.255], p = 0.048), long-term delayed recall (β = 0.89, 95% CI [0.198, 1.582], p = 0.012), cued recall (β = 1.085, 95% CI [0.414, 1.757], p = 0.002), and word recognition (β = 2.438, 95% CI [1.273, 3.603], p < 0.001) at immediate post-intervention. The intervention group also demonstrated a significant reduction in the severity of neuropsychic symptoms (NPS) (β = -2.166, 95% CI [-3.814, -0.519], p = 0.01). No significant changes were observed on cognitive shifting, working memory, response inhibition, attention, processing speed, the number of NPS, caregivers’ distress of NPS, functional ability, and HRQoL. In conclusion, CA-MECT was effective in improving cognition and reducing NPS among PwMD. It is warranted to examine the longer-term effects of the CA-MECT intervention.

